# Ten-Year Trends in the Use of Oral Anticoagulants in Australian General Practice Patients With Atrial Fibrillation

**DOI:** 10.3389/fphar.2021.586370

**Published:** 2021-03-23

**Authors:** Woldesellassie M. Bezabhe, Luke R. Bereznicki, Jan Radford, Barbara C. Wimmer, Colin Curtain, Mohammed S. Salahudeen, Gregory M. Peterson

**Affiliations:** ^1^ School of Pharmacy and Pharmacology, University of Tasmania, Hobart, TAS, Australia; ^2^ Launceston Clinical School, Tasmanian School of Medicine, University of Tasmania, Hobart, TAS, Australia

**Keywords:** trends, anticoagulants, atrial fibrillation, general practice, primary care, Australia

## Abstract

**Objective:** Appropriate use of oral anticoagulants (OACs) reduces the risk of stroke in patients with atrial fibrillation (AF). The study characterized the prescribing of OACs in people with AF in the Australian primary care setting over 10 years.

**Design:** Retrospective population study.

**Setting and Participants:** We performed 10 sequential cross-sectional analyses of patients with a recorded diagnosis of AF between 2009 and 2018 using national general practice data. The proportion of patients with AF who were prescribed an OAC based on their stroke risk was examined.

**Primary and secondary outcomes:** The primary outcome was the proportion of high stroke risk patients who were prescribed an OAC over a decade. The secondary outcome was variation in OAC prescribing among general practices.

**Results:** The sample size of patients with AF ranged from 9,874 in 2009 to 41,751 in 2018. The proportion who were prescribed an OAC increased from 39.5% (95% CI 38.6–40.5%) in 2009 to 52.0% (95% CI 51.5–52.4%) in 2018 (*p* for trend < 0.001). During this time, the proportion of patients with AF and high stroke risk who were prescribed an OAC rose from 41.7% (95% CI 40.7–42.8%) to 55.2% (95% CI 54.7–55.8%; *p* for trend < 0.001) with the direct-acting oral anticoagulants accounting for over three-quarters of usage by 2018. There was substantial variation in OAC prescribing between general practices. In 2018, the proportion of moderate to high stroke risk patients who were prescribed an OAC was 38.6% (95% CI 37.2–40.1%) in the lowest practice site quintiles and 65.6% (95% CI 64.5–66.7%) in the highest practice site quintiles.

**Conclusions:** Over the 10 years, OAC prescribing in high stroke risk patients with AF increased by one-third. There was considerable variation in OAC prescribing between general practices.

## Background

Appropriate utilization of oral anticoagulants reduces stroke risk in patients with atrial fibrillation (Aguilar and Hart, 2005). The vitamin K antagonist, warfarin, has been the mainstay of anticoagulation in AF for over 2 decades. It decreases the risk of stroke by almost two-thirds (Aguilar and Hart, 2005). However, it has a narrow therapeutic index and is associated with problematic drug and food interactions that require monitoring and dose adjustments. The direct-acting oral anticoagulants (DOACs) are at least non-inferior to warfarin in efficacy and safety (Connolly et al., 2009; Granger et al., 2011; Patel et al., 2011). In Australia, three DOACs (rivaroxaban, dabigatran, and apixaban) were listed for Commonwealth subsidy under the Pharmaceutical Benefits Scheme (PBS) for non-valvular AF in 2013; since then their overall use has markedly increased (Drug Utilisation Sub-Committee (DUSC), 2016; Admassie et al., 2017; Alamneh et al., 2017; Pol et al., 2018). In contrast, the prescribing of warfarin has declined (Drug Utilisation Sub-Committee (DUSC), 2016; Admassie et al., 2017; Alamneh et al., 2017; Pol et al., 2018).

Recent studies on the utilization of OAC have highlighted both underuse and overuse in patients with AF in Australia (Admassie et al., 2017; Alamneh et al., 2017; Schaffer et al., 2019). The Tasmanian AF Study observed prescribing practice from 2011 to 2015, and reported that 55 and 63% of eligible AF patients with a high stroke risk were prescribed an OAC before and after DOACs were listed on the PBS, respectively (Admassie et al., 2017). This study, however, involved only hospitalized patients, who might have been more co-morbid than those managed in primary care; the results therefore may not have reflected OAC prescribing rates in general practices nationally. The current AF prescribing patterns, in relation to stroke risk, in the Australian primary care setting remain unknown.

The primary objective of this study was to investigate the proportion of Australian primary care patients with AF prescribed an OAC according to their stroke risk, and temporal trends in prescribing patterns over a 10 year period. The secondary objective was to examine variation in OAC prescribing between general practices.

## Methods

Data for this study was obtained from NPS MedicineWise’s dataset, MedicineInsight. This is the largest and the most representative (in terms of gender, age, socioeconomic status) general practice dataset available to researchers in Australia (Busingye et al., 2019; MedicineInsight, 2020). A total of 429 practices sites contributed data for this study.

MedicineInsight uses a third-party tool that extracts, de-identifies and securely transmits patient data each week to its secure data repository. The extraction tool allows developing a longitudinal database of patients in general practices. The data that MedicineInsight collects from general practices include patient demographics, diagnoses, pathology test results, prescribed medications, and reasons for encounter. However, specific patient identifiers, such as patient name, address, and date of birth, are not included in this dataset (MedicineInsight, 2020).

We performed 10 sequential cross-sectional analyses of data on 1 September every year (census date) from September 01, 2009 to September 01, 2018. Patients with a recorded diagnosis of non-valvular AF were included in each analysis if 1) they were aged 18 years or older and not deceased on or before the census date, 2) they had had three or more recorded general practice visits in the previous two years and at least one of these visits was in the last six months, and 2) they had been registered in the general practice’s electronic records at least one year before the census date. We excluded patient who had a recorded OAC prescription before the diagnosis of AF. We defined patients with AF as being prescribed an OAC (warfarin, dabigatran, rivaroxaban or apixaban) or antiplatelet agent (clopidogrel, ticagrelor, aspirin, ticlopidine, prasugrel, dipyridamole, abciximab, eptifibatide or tirofiban) when they had at least one recorded prescription, dated within 365 days before the census date. The prescriptions recorded in this dataset were only those prescribed by general practitioners (GPs). Aspirin is available without a prescription, but we could only capture prescribed data.

For most of our study period, guidelines recommended using the CHA_2_DS_2_-VASc score (congestive heart failure (1 point), hypertension (1 point), age ≥75 years (2 points), diabetes mellitus (1 point), stroke/transient ischaemic attack (TIA) (2 points), vascular disease (1 point), age 65–74 years (1 point) and sex female (1 point)) for assessing stroke risk and treatment eligibility in patients with AF (Steffel et al., 2018). Current comorbidities and age at the census date were used to calculate CHA_2_DS_2_-VASc score. Current comorbidities were defined as those diagnosed and recorded on or before the census date. Patients with AF were stratified as low risk when CHA_2_DS_2_-VASc was 0 and male or one and female, moderate risk with CHA_2_DS_2_-VASc = 1 and male, and high risk with CHA_2_DS_2_-VASc ≥2 (Steffel et al., 2018). The proportion of patients who were prescribed an OAC, antiplatelet alone, or neither were calculated with 95% confidence interval (CI) each year on 1 September from September 01, 2009 through September 01, 2018. Temporal trends were shown using graphs and a Cochran-Armitage test for trend (Lachin, 2011) was used to determine if any observed trends were statistically significant.

Similarly, the proportion of patients with moderate to high stroke risk (CHA_2_DS_2_-VASc ≥ 1 and male or CHA_2_DS_2_-VASc ≥ 2) or low stroke risk (CHA_2_DS_2_-VASc = 0 for male or CHA_2_DS_2_-VASc = 1 for female) who were prescribed an OAC was calculated each year for each practice site. Potentially appropriate prescribing was defined as prescribing of an OAC to patients with a medium to high stroke risk. Potentially inappropriate prescribing was defined as prescribing an OAC to patients with low stroke risk. All practice sites that contributed data at least for a year were included. Prescribing rates were ranked into quintiles and used as an indicator of general practice sites’ prescribing performance. The variation between the highest- and lowest-prescribing practice quintiles each year was calculated as a prescribing gap. We calculated linear-weighted kappa coefficients for ordered categories to determine whether practice performance remained constant over the study period (Vanbelle and Albert, 2009).

Socio-economic indexes for areas (SEIFA) quintile is an index developed by the Australian Bureau of Statistics (ABS) and ranks areas in Australia from 1 (most disadvantaged area) to 5 (most advantaged area) (Australian Bureau of Statistics, 2018). The ABS categorize rurality into five categories using the Accessibility/Remoteness Index of Australia (ARIA) score. These categories are major cities (ARIA 0–0.20), inner regional (0.21–2.40), outer regional (2.41–5.92), remote (5.93–10.53), and very remote (10.54–15) (Australian Statistical Geography Standard (ASGS), 2017); we collapsed remote and very remote areas into one group. SAS software (SAS version 9.4, SAS Institute Inc., Cary, NC, United States) was used for all data analyses, and a two-sided *p*-value < 0.05 was considered statistically significant.

Ethics approval was obtained from the Tasmanian Health and Medical Human Research Ethics Committee (H0017648). We also obtained approval to conduct this study from MedicineInsight’s independent Data Governance Committee (2018–033). Patients were not identifiable, and individual patient consent was waived for our ethics application.

### Patient and Public Involvement

No patient involved.

## Results

### Baseline Characteristics

The total number of patients with AF included in our consecutive cross-sectional analyses ranged from 9,874 from 169 practice sites in 2009 to 41,751 from 429 practice sites in 2018. The mean age (standard deviation) of patients with AF increased slightly from 75.1 (11.6) years in 2009 to 76.0 (11.6) years in 2018 (*p* for trend < 0.001). The proportion of male patients increased from 51.4% (95% CI 50.4–52.4%) in 2009 to 54.5% (95% CI 54.0–55.0%) in 2018 (*p* for trend <0.001; Table 1).

**TABLE 1:** Demographic characteristics of patients with atrial fibrillation, 2009–2018.

Year	2009	2010	2011	2012	2013	2014	2015	2016	2017	2018
Sample (n)	9,874	13,723	17,807	22,510	26,777	32,285	35,641	38,804	41,338	41,751
Age (mean (SD)	75.1 (11.6)	75.3 (11.7)	75.3 (11.8)	75.7 (11.9)	75.3 (11.9)	75.3 (12.0)	75.5 (11.9)	75.6 (11.8)	75.7 (11.8)	76.0 (11.6)
Sex—male (%)	5,076 (51.4)	7,146 (52.1)	9,363 (52.6)	11,758 (52.2)	14,251 (53.2)	17,226 (53.4)	19,119 (53.6)	20,903 (53.9)	22,301 (54.0)	22,700 (54.4)
Indigenous status (%)										
ATSI	69 (0.7)	119 (0.9)	178 (1.0)	230 (1.0)	277 (1.0)	343 (0.6)	398 (1.1)	452 (1.2)	509 (1.2)	540 (1.3)
Non-ATSI	6,359 (64.4)	9,030 (65.8)	12,549 (70.5)	16,313 (72.5)	20,732 (77.4)	25,530 (79.1)	28,974 (81.3)	32,190 (83.0)	34,777 (84.1)	35,442 (84.9)
Missing	3,446 (34.9)	4,574 (33.3)	5,080 (28.5)	5,967 (26.5)	5,768 (21.5)	6,412 (19.9)	6,269 (17.6)	6,162 (15.9)	6,052 (14.6)	5,769 (13.8)
State (%)										
NSW	4,031 (40.8)	5,523 (40.3)	7,564 (42.5)	9,472 (42.1)	10,944 (40.9)	13,201 (40.9)	14,267 (40.0)	15,328 (39.5)	16,245 (39.3)	16,497 (39.5)
VIC	2,060 (20.9)	3,191 (23.3)	4,117 (23.1)	5,442 (24.2)	5,996 (22.4)	7,067 (21.9)	7,672 (21.5)	8,260 (21.3)	8,533 (20.6)	8,000 (19.2)
QLD	1,350 (13.7)	1,885 (13.7)	2,306 (13.0)	2,897 (12.9)	3,869 (14.5)	4,705 (14.6)	5,487 (15.4)	5,992 (15.4)	6,701 (16.2)	7,030 (16.8)
WA	878 (8.9)	1,054 (7.7)	1,188 (6.7)	1,348 (6.0)	2,055 (7.7)	2,765 (8.6)	3,163 (8.9)	3,659 (9.4)	4,002 (9.7)	4,102 (9.8)
TAS	1,208 (12.2)	1,511 (11.0)	1,729 (9.7)	2,156 (9.6)	2,311 (8.6)	2,727 (8.5)	2,937 (8.2)	3,064 (7.9)	3,209 (7.8)	3,454 (8.3)
SA	230 (12.2)	319 (2.3)	534 (3.0)	774 (3.4)	1,054 (3.9)	1,183 (3.7)	1,294 (3.6)	1,345 (3.5)	1,393 (3.4)	1,421 (3.4)
ACT	42 (0.4)	100 (0.7)	126 (0.7)	147 (0.7)	272 (1.0)	351 (1.1)	493 (1.4)	812 (2.1)	874 (2.1)	884 (2.1)
NT	75 (0.8)	140 (1.0)	243 (1.4)	274 (1.2)	276 (1.0)	286 (0.9)	328 (0.9)	344 (0.9)	381 (0.9)	363 (0.9)
Rurality (%)										
Major cities	5,189 (52.6)	7,355 (53.6)	9,460 (53.1)	12,237 (54.4)	15,167 (56.6)	18,469 (57.2)	20,528 (57.6)	22,581 (58.2)	24, 251 (58.7)	23,918 (57.3)
Inner regional	3,178 (32.2)	4,156 (30.3)	5,599 (31.4)	7,027 (31.2)	8,014 (29.9)	9,669 (30.0)	10,583 (29.7)	11,414 (29.4)	12,002 (29.0)	12,686 (30.4)
Outer regional	1,340 (13.6)	1,968 (14.3)	2,465 (13.8)	2,917 (13.0)	3,228 (12.1)	3,624 (11.2)	3,928 (11.0)	4,123 (10.6)	4,307 (10.4)	4,317 (10.3)
Remote/very remote	86 (0.9)	137 (1.0)	171 (1.0)	190 (0.8)	221 (0.8)	340 (1.1)	413 (1.2)	481 (1.2)	585 (1.4)	642 (1.5)
Missing	81 (0.8)	107 (0.8)	112 (0.6)	139 (0.6)	147 (0.6)	183 (0.6)	189 (0.5)	205 (0.5)	193 (0.5)	188 (0.5)
SEIFA quintiles (%)										
1	2,177 (22.1)	3,030 (22.1)	3,810 (21.4)	4,500 (20.0)	5,020 (18.8)	5,828 (18.1)	6,375 (17.9)	6,847 (17.7)	7,113 (17.2)	7,022 (16.8)
2	1,702 (17.2)	2,238 (16.3)	3,233 (18.2)	4,183 (18.6)	5,032 (18.8)	6,213 (19.2)	7,027 (19.7)	7,690 (19.8)	8,227 (19.9)	8,376 (20.1)
3	2,622 (26.6)	3,571 (26.0)	4,613 (25.9)	5,839 (25.9)	6,911 (25.8)	8,287 (25.7)	8,991 (25.2)	9,560 (24.6)	10,231 (24.8)	10,498 (25.1)
4	1,466 (14.9)	2,014 (14.7)	2,612 (14.7)	3,332 (14.8)	4,177 (15.6)	5,085 (15.8)	5,731 (16.1)	6,410 (16.5)	7,010 (17.0)	7,177 (17.2)
5	1,815 (18.4)	2,747 (20.0)	3,398 (19.1)	4,480 (19.9)	5,446 (20.3)	6,622 (20.5)	7,254 (20.4)	8,017 (20.7)	8,489 (20.5)	8,414 (20.2)
Missing	92 (0.9)	123 (0.9)	141 (0.8)	176 (0.8)	191 (0.7)	250 (0.8)	263 (0.7)	280 (0.7)	268 (0.7)	264 (0.6)
CHA_2_DS_2_-VASc score (%)										
Low (0 males, 1 in females)	548 (5.6)	805 (5.9)	1,072 (6.0)	1,349 (6.0)	1,753 (6.6)	2,155 (6.7)	2,345 (6.6)	2,569 (6.6)	2,713 (6.6)	2,548 (6.1)
Moderate (1 in males)	619 (6.3)	863 (6.3)	1,115 (6.3)	1,360 (6.0)	1,847 (6.9)	2,196 (6.8)	2,400 (6.7)	2,603 (6.7)	2,852 (6.9)	2,918 (7.0)
High (≥2)	8,707 (88.2)	12,055 (87.9)	15,620 (87.7)	19,801 (88.0)	23,177 (86.6)	27,934 (86.5)	30,896 (86.7)	33, 632 (86.7)	35,773 (86.5)	36,285 (87.0)

ATSI, Aboriginal and Torres Strait Islander; SD, standard deviation; SEIFA, socioeconomic indexes for areas.

### Oral Anticoagulant Prescribing

**FIGURE 1:**
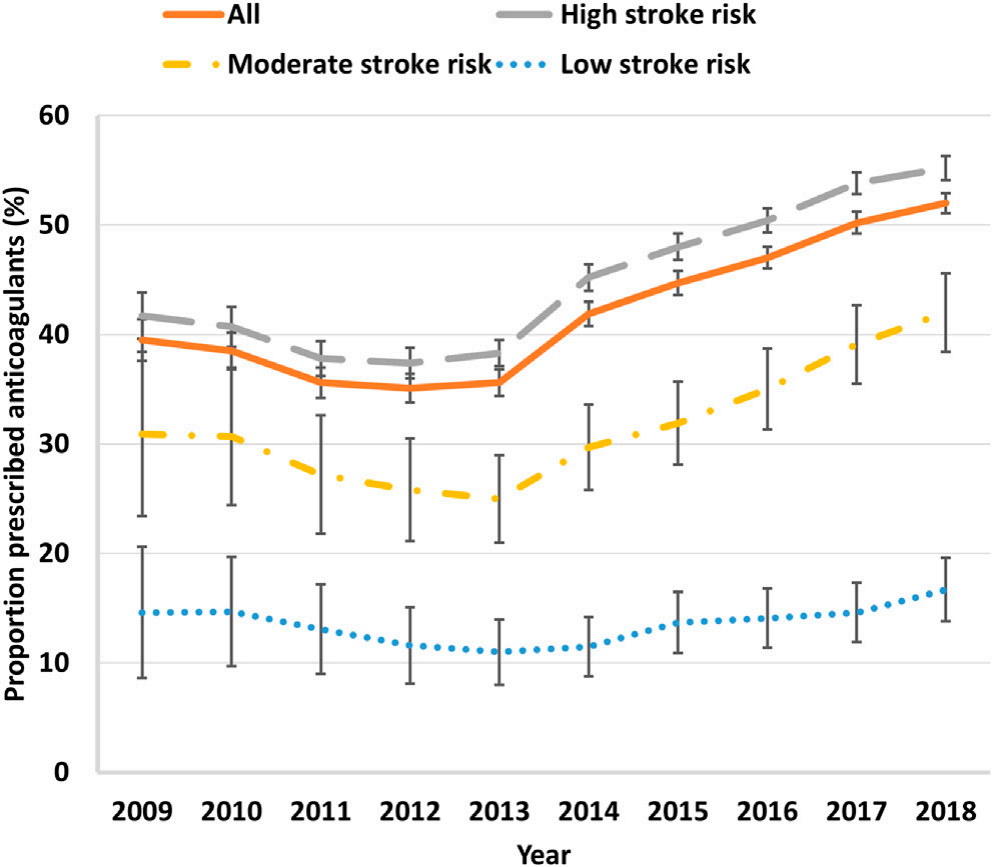
Trends of oral anticoagulant prescribing in Australian general practice patients with atrial fibrillation stratified by CHA2DS2-VASc score, 2009–2018. Error bars indicate 95% confidence intervals.

The proportion of patients with AF and an OAC prescription recorded decreased from 39.5% (95% CI 38.6–40.5%) in 2009 to 35.1% (95% CI 34.5–35.8%) in 2011 and then increased to 52.0% (95% CI 51.5–52.4%) by 2018 (*p* < 0.001; Figure 1). In all patients with AF, lone antiplatelet prescribing dropped steadily from 17.6% (95% CI 16.8–18.3%) in 2009 to 2.9% (95% CI 2.7–3.0%) in 2018 (*p* for decrease over time <0.001; Supplementary Table S1). However, these latter data are unreliable as patients can obtain aspirin without a prescription. The proportion of people who had no record of a prescription for either treatment to prevent stroke increased from 42.9% (95% CI 41.9–43.9%) to 51.1% (95% CI 50.5–51.7%) in 2013 and plateaued around 47.0% between 2014 and 2016, and then declined to 45.2% (95% CI 44.7–45.7%) in 2018 (*p* for increase over time <0.001; Supplementary Table S1).

In high-risk patients (CHA_2_DS_2_-VASc≥2), the proportion with an OAC prescription recorded increased from 41.7% (95% CI 40.7–42.8%) in 2009 to 55.2% (95% CI 54.7–55.8%) in 2018 (*p* < 0.001). In moderate stroke risk patients (CHA_2_DS_2_-VASc = 1 and male), the proportion who were receiving an OAC increased from 30.9% (95% CI 27.2–34.7%) in 2009 to 42.0% (95% CI 40.2–43.8%) in 2018 (*p* < 0.001). In low stroke risk patients with AF (CHA_2_DS_2_-VASc = 1 and female, 0 and male), the proportion who were prescribed an OAC decreased from 14.6% (95% CI 11.8–17.8%) in 2009 to 11.0% (95% CI 9.6–12.6%) in 2013 and then increased to 16.7% (95% CI 15.3–18.2%) in 2018 (*p* < 0.001; Figure 1).

### General Practices’ Prescribing Performance Gap Over Time

**FIGURE 2:**
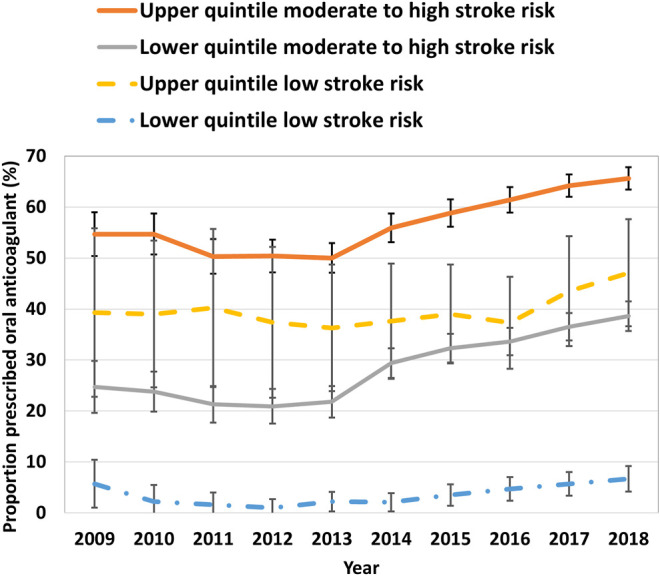
Practice site prescribing-performance quintile in moderate to high stroke risk patients with atrial fibrillation, 2009–2018. Error bars indicate 95% confidence intervals.

In 2009, the proportion of moderate to high stroke risk patients (CHA_2_DS_2_-VASc ≥ 1 and male or CHA_2_DS_2_-VASc ≥ 2 and female) with AF and an OAC prescription recorded among the lowest prescribing practice quintile was 24.7% (95% CI 22.3–27.4%), compared with 54.7% (95% CI 52.6–56.9%) in the highest quintile. By 2018, prescribing had increased to 38.6% (95% CI 37.2–40.1%) and 65.6% (95% CI 64.5–66.7%) in the lowest and highest practice quintiles, respectively. The gap between the highest- and lowest-prescribing practice quintiles in OAC prescribing for patients with moderate to high stroke risk remained wide, falling slightly from 30.0% in 2009 to 25.9% in 2018 (Figure 2).

A total of 429 practice sites contributed data in 2018. Of these, 169 (39.4%) had provided data since 2009, of which 64 (37.9%) of practice sites’ OAC prescribing quintile did not change, and 120 (71.0%) practice sites continued in the same or closest prescribing quintile. There was reasonable agreement in practices sites’ prescribing quintile between 2009 and 2018, weighted kappa = 0.34 (95% CI 0.24–0.45) (McHugh, 2012).

In 2009, the proportion of patients with AF who were prescribed an OAC while potentially not recommended (CHA_2_DS_2_-VASc = 0 and male or CHA_2_DS_2_-VASc = 1 and female) in the lowest- and highest-prescribing quintiles were 5.7% (95% CI 3.7–8.4%) and 39.3% (95% CI 31.3–47.8%), respectively. At the end of the study period, the proportion of potentially inappropriate prescribing in the lowest- and highest-prescribing quintiles had increased to 6.7% (95% CI 5.5–8.0%) and 47.1% (95% CI 41.9–52.4%), respectively (Figure 2).

### Trends in the Use of Warfarin and DOACs

**FIGURE 3:**
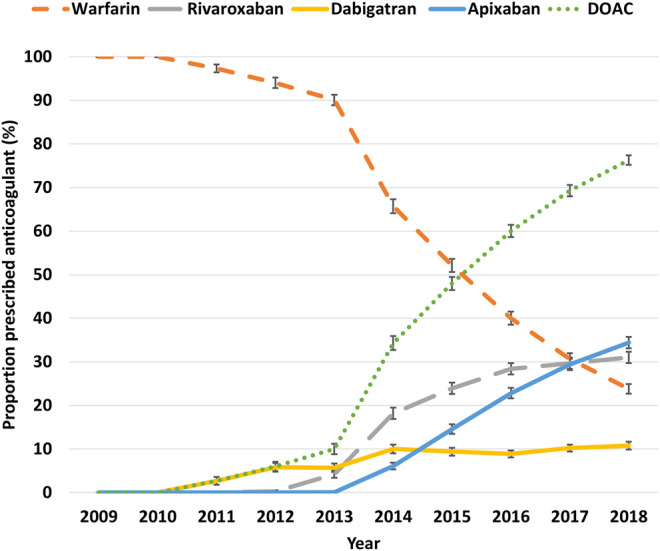
The proportion of patients with atrial fibrillation receiving an oral anticoagulant, 2009–2018. Error bars indicate 95% confidence intervals.

Among all patients with an OAC prescription recorded, the proportion who were prescribed a DOAC increased rapidly from 2.7% (95% CI 2.4–3.3%) in 2011 to 76.3% (95% CI 75.7–76.8%) in 2018, while the proportion of those prescribed warfarin correspondingly decreased from 97.3% (95% CI 96.8%97.7%) to 23.8% (95% CI 23.2–24.3%) (Figure 3; Supplementary Table S2).

## Discussion

The analyses of this large and nationally representative data suggest changing practice trends in the rate and type of OAC prescribing over the 10 year period. The proportion of patients with moderate to high stroke risk who were prescribed an OAC increased steadily by one-third from 2009 to 2018. This increase in the proportion patients with moderate to high stroke risk who were prescribed an OAC was significantly higher from 2013 onwards, corresponding with the PBS listing of DOACs for Australian government subsidization (rivaroxaban in August 2013, and apixaban and dabigatran in September 2013) (Drug Utilisation Sub-Committee (DUSC), 2016). In 2010, the European Society of Cardiology (ESC) guidelines recommended prescribing of an OAC for all AF patients at moderate-high risk of stroke, (i.e., CHA_2_DS_2_-VASc score ≥1) instead of antiplatelet therapy (Camm et al., 2010). This was followed by the 2012 ESC’s updated recommendation to avoid prescribing of aspirin in low-stroke risk patients (Camm et al., 2012). These changes may also explain the surge in OAC prescribing during the study period (Camm et al., 2012). Similar trends of an increase in OAC use, with a slow initial uptake after the introduction of DOACs, have been reported by studies from the United Kingdom and Denmark (Gadsbøll et al., 2017; Loo et al., 2017).

In 2018, just over half of the high-risk patients were prescribed an OAC. This rate is low compared with the rates reported from previous studies. The Tasmanian AF study found 63% of high-risk patients were prescribed an OAC. However, that study involved hospitalized patients who might have been more comorbid than general practice patients and it excluded patients with known OAC contraindications. A study in the United Kingdom using general practice data found that over three-quarters of high-risk patients with AF were prescribed an OAC (Adderley et al., 2018). Another study from Denmark found that two-thirds of patients were prescribed an OAC (Gadsbøll et al., 2017).

Despite an overall increase in OAC prescribing over the study period, there remained wide gaps between the highest- and lowest-performing practices in both appropriate (for moderate to high stroke risk) and potentially inappropriate (for low stroke risk) prescribing. One possible reason for the observed gaps in the appropriate use of an OAC might be the absence of regular reassessment of CHA_2_DS_2_-VASc scores. A study by Yoon et al. (Yoon et al., 2018) found that 46.6% of low-risk and 72% of moderate-risk patients at baseline were reclassified as being at high stroke risk within 10 years of follow-up. Increasing GPs’ awareness of the need for annual stroke risk assessment may improve OAC prescribing.

## Strengths and Limitations

This was the first AF study conducted using MedicineInsight dataset, which provided a large and national study population and thus enabled a comprehensive description of GP prescribing of OACs in Australia (González-Chica et al., 2018; Radford et al., 2018; MedicineInsight, 2020). Furthermore, 10 years sequential cross-sectional analyses enabled characterizing the longitudinal trends in OAC prescribing.

The study has several limitations. The MedicineInsight dataset contains only records of medications prescribed by GPs. However, GPs in Australia typically continue those medications prescribed by cardiologists and so the trends described in this study may still be considered accurate and useful with regard to overall OAC prescribing. We did not account for medication contraindications and adverse drug reactions, that may have prevented GPs from prescribing an OAC.

In this study, we used the guidelines retrospectively. For instance, before 2012, OAC treatment was recommended for patients at moderate to high stroke risk, and aspirin was widely used for patients at low stroke risk (Camm et al., 2010). However, the guidelines used for this analysis were in use for most of the study period and are appropriate to evaluate the trends.

## Conclusion

Over the 10 years, overall OAC prescribing increased by one-third. By 2018, 55.2% of the patients with a high stroke risk had an OAC prescription recorded, with the proportion varying substantially between practices. There remains scope to improve OAC prescribing for AF in the primary care setting, and the reasons for withholding OAC therapy in eligible patients need to be investigated.

## Data Availability

The datasets presented in this article are not readily available because the data analyzed in this study was obtained from MedicineInsight with the restriction of not sharing the data publicly. Requests to access these datasets should be directed to MedicineInsight, DataGovernance@nps.org.au. Requests to access the datasets should be directed to DataGovernance@nps.org.au.
