# Supplementary material for: Ten-Year Trends in the Use of Oral Anticoagulants in Australian General Practice Patients With Atrial Fibrillation
Source: Front Pharmacol. 2021 Mar 23;12:586370. doi: 10.3389/fphar.2021.586370 (PMC8044929; doi:10.3389/fphar.2021.586370)
Supplement: Supplementary file 1 [file datasheet1.docx]

Table S1. Trends in oral anticoagulant prescribing in Australian general patients with AF, 2009-2018.

| Year | CHA_2_DS_2_-VASc score | Population | | Prescribed OACs | | Prescribed Antiplatelets only | | Prescribed Neither Treatment | |
| --- | --- | --- | --- | --- | --- | --- | --- | --- | --- |
|  |  | n | % (95%CI) | n | % (95%CI) | n | % (95%CI) | n | % (95%CI) |
| 2009 | All | 9,874 |  | 3,903 | 39.5 (38.6-40.5) | 1,734 | 17.6 (16.8-18.3) | 4,237 | 42.9 (41.9-43.9) |
|  | High | 8,707 | 88.2 (87.5-88.8) | 3,632 | 41.7 (40.7-42.8) | 1,608 | 18.5 (17.7-19.3) | 3,467 | 39.8 (38.8-40.9) |
|  | Moderate | 619 | 6.3 (5.8-6.8) | 191 | 30.9 (27.2-34.7) | 85 | 13.7 (11.1-16.7) | 343 | 55.4 (51.4-59.4) |
|  | Low | 548 | 5.6 (5.1-6.0) | 80 | 14.6 (11.8-17.8) | 41 | 7.5 (5.4-10.0) | 427 | 77.9 (74.2-81.3) |
| 2010 | All | 13,723 |  | 5,289 | 38.5 (37.7-39.4) | 2,307 | 16.8 (16.2-17.5) | 6,127 | 44.7 (43.8-45.5) |
|  | High | 12,055 | 87.9 (87.3-88.4) | 4,906 | 40.7 (39.8-41.6) | 2,141 | 17.8 (17.1-18.5) | 5,008 | 41.5 (40.7-42.4) |
|  | Moderate | 863 | 6.3 (5.9-6.7) | 265 | 30.7 (27.6-33.9) | 105 | 12.2 (10.1-14.5) | 493 | 57.1 (53.8-60.5) |
|  | Low | 805 | 5.9 (5.5-6.3) | 118 | 14.7 (12.3-17.3) | 61 | 7.6 (5.9-9.6) | 626 | 77.8 (74.7-80.6) |
| 2011 | All | 17,807 |  | 6,345 | 35.6 (34.9-36.3) | 2,821 | 15.8 (15.3-16.4) | 8,641 | 48.5 (47.8-49.3) |
|  | High | 15,620 | 87.7 (87.2-88.2) | 5,902 | 37.8 (37.0-38.6) | 2,606 | 16.7 (16.1-17.3) | 7,112 | 45.5 (44.6-46.3) |
|  | Moderate | 1,115 | 6.3 (5.9-6.6) | 303 | 27.2 (24.6-30.0) | 135 | 12.1 (10.3-14.2) | 677 | 60.7 (57.8-63.6) |
|  | Low | 1,072 | 6.0 (5.7-6.4) | 140 | 13.1 (11.1-15.2) | 80 | 7.5 (6.0-9.2) | 852 | 79.5 (76.9-81.9) |
| 2012 | All | 22, 510 |  | 7,910 | 35.1 (34.5-35.8) | 3,139 | 13.9 (13.5-14.4) | 11,461 | 50.9 (50.3-51.6) |
|  | High | 19,801 | 88.0 (87.5-88.4) | 7,403 | 37.4 (36.7-38.1) | 2,902 | 14.7 (14.2-15.2) | 9,496 | 48.0 (47.3-48.7) |
|  | Moderate | 1,360 | 6.0 (5.7-6.4) | 351 | 25.8 (23.5-28.2) | 144 | 10.6 (9.0-12.4) | 865 | 63.6 (61.0-66.2) |
|  | Low | 1,349 | 6.0 (5.7-6.3) | 156 | 11.6 (9.9-13.4) | 93 | 6.9 (5.6-8.4) | 1,100 | 81.5 (79.4-83.6) |
| 2013 | All | 26,777 |  | 9,533 | 35.6 (35.0-36.2) | 3,560 | 13.3 (12.9-13.7) | 13,684 | 51.1 (50.5-51.7) |
|  | High | 23, 177 | 86.6 (86.1-87.0) | 8,878 | 38.3 (37.7-38.9) | 3,270 | 14.1 (13.7-14.6) | 11,029 | 47.6 (46.9-48.2) |
|  | Moderate | 1,847 | 6.9 (6.6-7.2) | 462 | 25.0 (23.1-27.1)) | 192 | 10.4 (9.0-11.9) | 1,193 | 64.6 (62.4-66.7) |
|  | Low | 1,753 | 6.6 (6.3-6.9) | 193 | 11.0 (9.6-12.6) | 98 | 5.6 (4.6-6.8) | 1,462 | 83.4 (81.6-85.1) |
| 2014 | All | 32, 285 |  | 13,530 | 41.9 (41.4-42.5) | 3,517 | 10.9 (10.6-11.2) | 15,238 | 47.2 (46.7-47.8) |
|  | High | 27,934 | 86.5 (86.2-86.9) | 12,630 | 45.2 (44.6-45.8) | 3,225 | 11.6 (11.2-11.9) | 12,079 | 43.2 (42.7-43.8) |
|  | Moderate | 2,196 | 6.8 (6.5-7.1) | 652 | 29.7 (27.8-31.7) | 202 | 9.2 (8.0-10.5) | 1,342 | 61.1 (59.0-63.2) |
|  | Low | 2,155 | 6.7 (6.4-7.0) | 248 | 11.5 (10.2-12.9) | 90 | 4.2 (3.4-5.1) | 1,817 | 84.3 (82.7-85.8) |
| 2015 | All | 35,641 |  | 15,912 | 44.7 (44.1-45.2) | 2,920 | 8.2 (7.9-8.5) | 16,809 | 47.2 (46.6-47.7) |
|  | High | 30,896 | 86.7 (86.3-87.0) | 14,829 | 48.0 (47.4-48.6) | 2,698 | 8.7 (8.4-9.1) | 13,369 | 43.3 (42.7-43.8) |
|  | Moderate | 2,400 | 6.7 (6.5-7.0) | 765 | 31.9 (30.0-33.8) | 153 | 6.4 (5.4-7.4) | 1,482 | 61.8 (59.8-63.7) |
|  | Low | 2,345 | 6.6 (6.3-6.8) | 318 | 13.7 (12.2-15.0) | 69 | 2.9 (2.3-3.7) | 1,958 | 83.5 (81.9-85.0) |
| 2016 | All | 38,804 |  | 18,229 | 47.0 (46.5-47.5) | 2,179 | 5.6 (5.4-5.9) | 18, 396 | 47.4 (46.9-47.9) |
|  | High | 33,632 | 86.7 (86.3-87.0) | 16,955 | 50.4 (49.9-51.0) | 2,008 | 6.0 (5.7-6.2) | 14,669 | 43.6 (43.1-44.2) |
|  | Moderate | 2,603 | 6.7 (6.5-7.0) | 912 | 35.0 (33.2-36.9) | 97 | 3.7 (3.0-4.5) | 1,594 | 61.2 (59.3-63.1) |
|  | Low | 2,569 | 6.6 (6.4-6.9) | 362 | 14.1 (12.8-15.5) | 74 | 2.9 (2.3-3.6) | 2,133 | 83.0 (81.5-84.5) |
| 2017 | All | 41,338 |  | 20,754 | 50.2 (49.7-50.7) | 1,752 | 4.2 (4.1-4.4) | 18,832 | 45.6 (45.1-46.0) |
|  | High | 35,773 | 86.5 (86.2-86.9) | 19,243 | 53.8 (53.3-54.3) | 1,612 | 4.5 (4.3-4.7) | 14,918 | 41.7 (41.2-42.2) |
|  | Moderate | 2,852 | 6.9 (6.7-7.2) | 1,115 | 39.1 (37.3-40.9) | 85 | 3.0 (2.4-3.7) | 1,652 | 57.9 (56.1-59.7) |
|  | Low | 2,713 | 6.6 (6.3-6.8) | 396 | 14.6 (13.3-16.0) | 55 | 2.0 (1.5-2.6) | 2,262 | 83.4 (81.9-84.8) |
| 2018 | All | 41,751 |  | 21,693 | 52.0 (51.5-52.4) | 1,190 | 2.9 (2.7-3.0) | 18,868 | 45.2 (44.7-45.7) |
|  | High | 36,285 | 86.9 (86.6-87.2) | 20,042 | 55.2 (54.7-55.8) | 1,102 | 3.0 (2.9-3.2) | 15, 141 | 41.7 (41.2-42.2) |
|  | Moderate | 2,918 | 7.0 (6.8-7.2) | 1,226 | 42.0 (40.2-43.8) | 51 | 1.8 (1.3-2.3) | 1,641 | 56.2 (54.4-58.1) |
|  | Low | 2,548 | 6.1 (5.9-6.3) | 425 | 16.7 (15.3-18.2) | 37 | 1.5 (1.0-2.0) | 2,086 | 81.9 (80.3-83.4) |
| AF, atrial fibrillation; OAC, oral anticoagulant | | | | | | | | | |

Table S2. Trends in OAC use in patients with AF stratified by the CHA_2_DS_2_-VASc score, 2009-2018.

| Year | CHA_2_DS_2_-VASc score | Population | | Warfarin | | DOAC | | Rivaroxaban | | Dabigatran | | Apixaban | |
| --- | --- | --- | --- | --- | --- | --- | --- | --- | --- | --- | --- | --- | --- |
|  |  | n | % (95%CI) | n | % (95%CI) | n | % (95%CI) | n | % (95%CI) | n | % (95%CI) | n | % (95%CI) |
| 2009 | All | 3,903 |  | 3,903 | 100 (99.9-100) | 0 | 0.0 | 0 | 0.0 | 0 | 0.0 | 0 | 0.0 |
|  | High | 3,632 | 93.1 (92.2-93.8) | 3,632 | 100 (99.9-100) | 0 | 0.0 | 0 | 0.0 | 0 | 0.0 | 0 | 0.0 |
|  | Moderate | 191 | 4.9 (4.2-5.6) | 191 | 100 (98.1-100) | 0 | 0.0 | 0 | 0.0 | 0 | 0.0 | 0 | 0.0 |
|  | Low | 80 | 2.1 (1.6-2.5) | 80 | 100 (95.5-100) | 0 | 0.0 | 0 | 0.0 | 0 | 0.0 | 0 | 0.0 |
| 2010 | All | 5,289 |  | 5,289 | 100 (99.9-100) | 0 | 0.0 | 0 | 0.0 | 0 | 0.0 | 0 | 0.0 |
|  | High | 4,906 | 92.8 (92.0-93.4) | 4,906 | 100 (99.9-100) | 0 | 0.0 | 0 | 0.0 | 0 | 0.0 | 0 | 0.0 |
|  | Moderate | 265 | 5.0 (4.4-5.6) | 265 | 100 (98.6-100) | 0 | 0.0 | 0 | 0.0 | 0 | 0.0 | 0 | 0.0 |
|  | Low | 118 | 2.2 (1.9-2.7) | 118 | 100 (96.9-100) | 0 | 0.0 | 0 | 0.0 | 0 | 0.0 | 0 | 0.0 |
| 2011 | All | 6,345 |  | 6,171 | 97.3 (96.8-97.7) | 174 | 2.7 (2.4-3.3) | 0 | 0.0 | 174 | 2.7 (2.4-3.3) | 0 | 0.0 |
|  | High | 5,902 | 93.0 (92.4-93.6) | 5,745 | 97.3 (96.9-97.7) | 157 | 2.7 (2.2-3.1) | 0 | 0.0 | 157 | 2.7 (2.2-3.1) | 0 | 0.0 |
|  | Moderate | 303 | 4.8 (4.3-5.3) | 290 | 95.7 (92.8-97.7) | 13 | 4.3 (2.3-7.2) | 0 | 0.0 | 13 | 4.3 (2.3-7.2) | 0 | 0.0 |
|  | Low | 140 | 2.2 (1.9-2.6) | 136 | 97.1 (92.8-99.2) | 4 | 2.9 (0.8-7.2) | 0 | 0.0 | 4 | 2.9 (0.8-7.2) | 0 | 0.0 |
| 2012 | All | 7,910 |  | 7,433 | 94.0 (93.4-94.5) | 477 | 6.0 (5.5-6.6) | 20 | 0.3 (0.2-0.4) | 457 | 5.8 (5.3-6.3) | 0 | 0.0 |
|  | High | 7,403 | 94.0 (93.0-94.1) | 6,951 | 93.9 (93.3-94.4) | 452 | 6.1 (5.6-6.7) | 20 | 0.3 (0.2-0.4) | 432 | 5.8 (5.3-6.4) | 0 | 0.0 |
|  | Moderate | 351 | 4.4 (4.0-4.9) | 331 | 94.3 (91.3-96.5) | 20 | 5.7 (3.5-8.7) | 0 | 0.0 | 20 | 5.7 (3.5-8.7) | 0 | 0.0 |
|  | Low | 156 | 2.0 (1.7-2.3) | 151 | 96.8 (92.7-99.0) | 5 | 3.2 (1.1-7.3) | 0 | 0 | 5 | 3.2 (1.1-7.3) | 0 | 0.0 |
| 2013 | All | 9,533 |  | 8,584 | 90.1 (89.4-90.6) | 949 | 10.0 (9.4-10.6) | 401 | 4.2 (3.8-4.6) | 543 | 5.7 (5.2-6.2) | 5 | 0.1 (0.0-0.1) |
|  | High | 8,878 | 93.1 (92.6-93.6) | 7,979 | 89.9 (89.2-90.5) | 899 | 10.1 (9.5-10.8) | 381 | 4.3 (3.9-4.7) | 513 | 5.8 (5.3-6.3) | 5 | 0.1 (0.0-0.1) |
|  | Moderate | 462 | 4.9 (4.4-5.3) | 422 | 91.3 (88.4-93.7) | 40 | 8.7 (6.3-11.6) | 16 | 3.5 (2.0-5.6) | 24 | 5.2 (3.4-7.6) | 0 | 0.0 |
|  | Low | 193 | 2.0 (1.8-2.3) | 183 | 94.8 (90.7-97.5) | 10 | 5.2 (2.5-9.3) | 4 | 2.1 (0.6-5.2) | 6 | 3.1 (1.2-6.6) | 0 | 0.0 |
| 2014 | All | 13,530 |  | 8,889 | 65.7 (64.9-66.5) | 4641 | 34.3 (33.5-35.1) | 2,467 | 18.2 (17.6-18.9) | 1,353 | 10.0 (9.5-10.5) | 821 | 6.1 (5.7-6.5) |
|  | High | 12,630 | 93.4 (92.9-93.8) | 8,341 | 66.0 (65.2-66.9) | 4289 | 34.0 (33.1-34.8) | 2,255 | 17.9 (17.2-18.5) | 1,262 | 10.0 (9.5-10.5) | 772 | 6.1 (5.7-6.5) |
|  | Moderate | 652 | 4.8 (4.5-5.2) | 385 | 59.1 (55.2-62.9) | 267 | 41.0 (37.2-44.8) | 162 | 24.9 (21.6-28.4) | 67 | 10.3 (8.1-12.9) | 38 | 5.8 (4.2-7.9) |
|  | Low | 248 | 1.8 (1.6-2.1) | 163 | 65.7 (59.5-71.6) | 85 | 34.3 (28.4-40.5) | 50 | 20.2 (15.4-25.7) | 24 | 9.7 (6.3-14.1) | 11 | 4.4 (2.2-7.8) |
| 2015 | All | 15,912 |  | 8,282 | 52.1 (51.3-52.8) | 7630 | 48.0 (47.2-48.7) | 3,807 | 23.9 (23.3-24.6) | 1,499 | 9.4 (9.0-9.9) | 2,324 | 14.6 (14.1-15.2) |
|  | High | 14,829 | 93.2 (92.8-93.6) | 7,790 | 52.5 (51.7-53.3) | 7039 | 47.5 (46.7-48.3) | 3,462 | 23.4 (22.7-24.0) | 1,406 | 9.5 (9.0-10.0) | 2,171 | 14.6 (14.1-15.2) |
|  | Moderate | 765 | 4.8 (4.5-5.2) | 338 | 44.2 (40.6-47.8) | 427 | 55.8 (52.2-59.4) | 249 | 32.5 (29.2-36.0) | 63 | 8.2 (6.4-10.4) | 115 | 15.0 (12.6-17.8) |
|  | Low | 318 | 2.0 (1.8-2.2) | 154 | 48.4 (42.8-54.1) | 164 | 51.6 (45.9-57.2) | 96 | 30.2 (25.2-35.6) | 30 | 9.4 (6.5-13.2) | 38 | 12.0 (8.6-16.0) |
| 2016 | All | 18,229 |  | 7,298 | 40.0 (39.3-40.8) | 10931 | 60.0 (59.3-60.7) | 5,168 | 28.4 (27.7-29.0) | 1,614 | 8.9 (8.5-9.3) | 4,149 | 22.8 (22.2-23.4) |
|  | High | 16,955 | 93.0 (92.6-93.4) | 6,866 | 40.5 (39.8-41.2) | 10089 | 59.5 (58.8-60.2) | 4,702 | 27.7 (27.1-28.4) | 1,512 | 8.9 (8.5-9.4) | 3,875 | 22.9 (22.2-23.5) |
|  | Moderate | 912 | 5.0 (4.7-5.3) | 294 | 32.2 (29.2-35.4) | 618 | 67.8 (64.6-70.8) | 340 | 37.8 (34.1-40.5) | 72 | 7.9 (6.2-9.8) | 206 | 22.6 (19.9-25.4) |
| 2017 | Low | 362 | 2.0 (1.8-2.2) | 138 | 38.1 (33.1-43.3) | 224 | 38.1 (56.7-66.9) | 126 | 34.8 (29.9-40.0) | 30 | 8.3 (5.7-11.6) | 68 | 18.8 (14.9-23.2) |
|  | All | 20,754 |  | 6,365 | 30.7 (30.0-31.3) | 14389 | 69.3 (68.7-70.0) | 6,170 | 29.7 (29.1-30.4) | 2,113 | 10.2 (9.8-10.6) | 6,106 | 29.4 (28.8-30.1) |
|  | High | 19,243 | 92.7 (92.4-93.1) | 6,012 | 31.2 (30.6-31.9) | 13231 | 68.8 (68.1-69.4) | 5,594 | 29.1 (28.4-29.7) | 1,964 | 10.2 (9.8-10.6) | 5,673 | 29.5 (28.8-30.1) |
|  | Moderate | 1,115 | 5.4 (5.1-5.7) | 253 | 22.7 (20.3-25.3) | 862 | 77.3 (74.7-79.7) | 431 | 38.7 (35.8-41.6) | 106 | 9.5 (7.9-11.4) | 325 | 29.2 (26.5-31.9) |
|  | Low | 396 | 1.9 (1.7-2.1) | 100 | 25.3 (21.1-29.8) | 296 | 74.8 (70.2-79.0) | 145 | 36.6 (31.9-41.6) | 43 | 10.7 (8.0-14.4) | 108 | 27.3 (22.9-31.9) |
| 2018 | All | 21,693 |  | 5,153 | 23.8 (23.2-24.3) | 16540 | 76.3 (75.7-76.8) | 6,733 | 31.0 (30.4-31.7) | 2,350 | 10.8 (10.4-11.3) | 7,457 | 34.4 (33.7-35.0) |
|  | High | 20,042 | 92.4 (92.0-92.7) | 4,854 | 24.2 (23.6-24.8) | 15188 | 75.8 (75.2-76.4) | 6,083 | 30.4 (29.7-31.0) | 2,174 | 10.9 (10.4-11.3) | 6,931 | 34.6 (33.9-35.3) |
|  | Moderate | 1,226 | 5.7 (5.4-6.0) | 222 | 18.1 (16.0-20.4) | 1004 | 81.9 (79.6-84.0) | 487 | 39.7 (37.0-42.5) |  | 10.4 (8.7-12.2) | 390 | 31.8 (29.2-34.5) |
|  | Low | 425 | 2.0 (1.8-2.2) | 77 | 18.1 (14.6-22.1) | 348 | 81.9 (77.9-85.4) | 163 | 38.4 (33.7-43.2) |  | 11.5 (8.7-15.0) | 136 | 32.0 (27.6-36.7) |
| AF, atrial fibrillation; OAC, oral anticoagulant; DOAC, direct-acting oral anticoagulant | | | | | | | | | | | | | |
